# Supplementary material for: Pre-Senescence Induction in Hepatoma Cells Favors Hepatitis C Virus Replication and Can Be Used in Exploring Antiviral Potential of Histone Deacetylase Inhibitors
Source: Int J Mol Sci. 2021 Apr 27;22(9):4559. doi: 10.3390/ijms22094559 (PMC8123837; doi:10.3390/ijms22094559)
Supplement: Supplementary file 1 [file ijms-22-04559-s001.zip › ijms-1070369-supplementary.pdf]

Figure S1

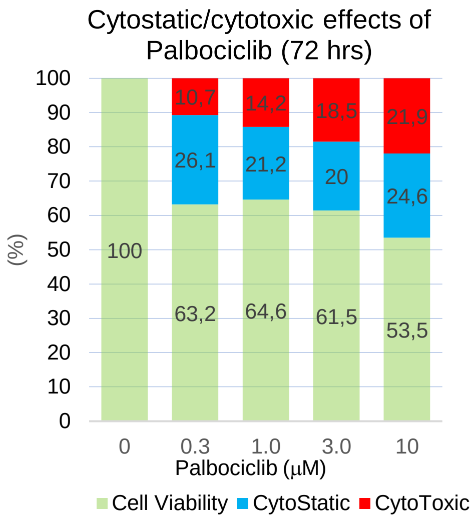

Figure S1. Evaluation of cytostatic/cytotoxic effects of palbociclib on Huh7-luc/neo cells. Percentages of damaged and alive cells were assessed by PI uptake and MTT colorimetric assay (CytoToxic and Cell Viability, correspondingly). The difference between this value and 100% was defined as a cytostatic effect (CytoStatic).
